# Supplementary material for: Biaxial strength under fatigue of 4YSZ: Surface treatment and resin cement viscosity effects
Source: PLoS One. 2026 May 28;21(5):e0349754. doi: 10.1371/journal.pone.0349754 (PMC13218516; doi:10.1371/journal.pone.0349754)
Supplement: Attachment — Submitted filename: Response (R1).docx [file pone.0349754.s003.docx]

**April-27-2026**

Manuscript Number: PONE-D-25-54706

Dear Prof. **Gaurav Arora**

*Academic Editor*

PLOS One

Thank you for considering our study for publication in the Journal PLOS One. We appreciated the opportunity and revised the manuscript according to the examinations. Comments and responses to the reviewer's suggestions are described below in red and the modifications have been highlighted (in yellow) in the manuscript.

We hope the changes and explanations are adequate. If during this new appreciation more revisions/explanations prove necessary, we are completely at your disposal.

Sincerely,

Dr. Tribst JPM

ACTA -Amsterdam

====================

====================

PONE-D-25-54706

Biaxial Strength under fatigue of 4YSZ: Surface Treatment and Resin Cement Viscosity effects

PLOS One

Dear Dr. Tribst,

Thank you for submitting your manuscript to PLOS ONE. After careful consideration, we feel that it has merit but does not fully meet PLOS ONE’s publication criteria as it currently stands. Therefore, we invite you to submit a revised version of the manuscript that addresses the points raised during the review process.

==============================
Major revision is suggested by the reviewers for the manuscript.
==============================

Please submit your revised manuscript by May 31 2026 11:59PM. If you will need more time than this to complete your revisions, please reply to this message or contact the journal office at [plosone@plos.org](mailto:plosone@plos.org). When you're ready to submit your revision, log on to <https://www.editorialmanager.com/pone/> and select the 'Submissions Needing Revision' folder to locate your manuscript file.

Please include the following items when submitting your revised manuscript:

- A letter that responds to each point raised by the academic editor and reviewer(s). You should upload this letter as a separate file labeled 'Response to Reviewers'.
- A marked-up copy of your manuscript that highlights changes made to the original version. You should upload this as a separate file labeled 'Revised Manuscript with Track Changes'.
- An unmarked version of your revised paper without tracked changes. You should upload this as a separate file labeled 'Manuscript'.

If you would like to make changes to your financial disclosure, please include your updated statement in your cover letter. Guidelines for resubmitting your figure files are available below the reviewer comments at the end of this letter.

If applicable, we recommend that you deposit your laboratory protocols in [protocols.io](http://protocols.io) to enhance the reproducibility of your results. Protocols.io assigns your protocol its own identifier (DOI) so that it can be cited independently in the future. For instructions see: [https://journals.plos.org/plosone/s/submission-guidelines#loc-laboratory-protocols](https://track.editorialmanager.com/CL0/https:%2F%2Fjournals.plos.org%2Fplosone%2Fs%2Fsubmission-guidelines%23loc-laboratory-protocols/1/010f019d99287204-4dee9e64-f943-43b6-a230-7258b23cedfb-000000/o4RWqCWPgoN9JUBlzeXrlR3fKKbW9XVKzO6YArZE3d8=258). Additionally, PLOS ONE offers an option for publishing peer-reviewed Lab Protocol articles, which describe protocols hosted on [protocols.io](http://protocols.io). Read more information on sharing protocols at [https://plos.org/protocols?utm_medium=editorial-email&utm_source=authorletters&utm_campaign=protocols](https://track.editorialmanager.com/CL0/https:%2F%2Fplos.org%2Fprotocols%3Futm_medium=editorial-email%26utm_source=authorletters%26utm_campaign=protocols/1/010f019d99287204-4dee9e64-f943-43b6-a230-7258b23cedfb-000000/4E7kClJVI5WH4znKKuS0r4lG1RKzN6q9d8iGem2xOPM=258).

As the corresponding author, your ORCID iD is verified in the submission system and will appear in the published article. PLOS supports the use of ORCID, and we encourage all coauthors to register for an ORCID iD and use it as well. Please encourage your coauthors to verify their ORCID iD within the submission system before final acceptance, as unverified ORCID iDs will not appear in the published article. Only the individual author can complete the verification step; PLOS staff cannot verify ORCID iDs on behalf of authors.

We look forward to receiving your revised manuscript.

Kind regards,

Gaurav Arora, Ph.D
Academic Editor
PLOS One

Journal Requirements:

When submitting your revision, we need you to address these additional requirements.

1. Please ensure that your manuscript meets PLOS ONE's style requirements, including those for file naming. The PLOS ONE style templates can be found at

<https://journals.plos.org/plosone/s/file?id=wjVg/PLOSOne_formatting_sample_main_body.pdf> and

[https://journals.plos.org/plosone/s/file?id=ba62/PLOSOne_formatting_sample_title_authors_affiliations.pdf](https://track.editorialmanager.com/CL0/https:%2F%2Fjournals.plos.org%2Fplosone%2Fs%2Ffile%3Fid=ba62%2FPLOSOne_formatting_sample_title_authors_affiliations.pdf/1/010f019d99287204-4dee9e64-f943-43b6-a230-7258b23cedfb-000000/gvN6v9nB4-05W_gvxYp7TNCB0brxKmBj8to-Pl6p7NA=258)

2. Please note that PLOS One has specific guidelines on code sharing for submissions in which author-generated code underpins the findings in the manuscript. In these cases, we expect all author-generated code to be made available without restrictions upon publication of the work. Please review our guidelines at <https://journals.plos.org/plosone/s/materials-and-software-sharing#loc-sharing-code> and ensure that your code is shared in a way that follows best practice and facilitates reproducibility and reuse.

3. We note that your Data Availability Statement is currently as follows: "

All relevant data are within the manuscript and its Supporting Information files."

Please confirm at this time whether or not your submission contains all raw data required to replicate the results of your study. Authors must share the “minimal data set” for their submission. PLOS defines the minimal data set to consist of the data required to replicate all study findings reported in the article, as well as related metadata and methods ([https://journals.plos.org/plosone/s/data-availability#loc-minimal-data-set-definition](https://track.editorialmanager.com/CL0/https:%2F%2Fjournals.plos.org%2Fplosone%2Fs%2Fdata-availability%23loc-minimal-data-set-definition/1/010f019d99287204-4dee9e64-f943-43b6-a230-7258b23cedfb-000000/6R2QVSfX7WmXnw-NVwd3FpkKqRpD-dFtUtA1xNUh1Xo=258)).

For example, authors should submit the following data:

- The values behind the means, standard deviations and other measures reported;

- The values used to build graphs;

- The points extracted from images for analysis.

Authors do not need to submit their entire data set if only a portion of the data was used in the reported study.

If your submission does not contain these data, please either upload them as Supporting Information files or deposit them to a stable, public repository and provide us with the relevant URLs, DOIs, or accession numbers. For a list of recommended repositories, please see [https://journals.plos.org/plosone/s/recommended-repositories](https://track.editorialmanager.com/CL0/https:%2F%2Fjournals.plos.org%2Fplosone%2Fs%2Frecommended-repositories/1/010f019d99287204-4dee9e64-f943-43b6-a230-7258b23cedfb-000000/JU_Bx-0Rh5uDP5UAt2dHU9t-765SCVeP2W9R9Qp3gg4=258).

If there are ethical or legal restrictions on sharing a de-identified data set, please explain them in detail (e.g., data contain potentially sensitive information, data are owned by a third-party organization, etc.) and who has imposed them (e.g., an ethics committee). Please also provide contact information for a data access committee, ethics committee, or other institutional body to which data requests may be sent. If data are owned by a third party, please indicate how others may request data access.

4. Please include a copy of Table 5 which you refer to in your text on page 21.

5. If the reviewer comments include a recommendation to cite specific previously published works, please review and evaluate these publications to determine whether they are relevant and should be cited. There is no requirement to cite these works unless the editor has indicated otherwise. 

We sincerely thank the editor for the consideration of our manuscript. We hope that our work will be considered suitable for publication in PLOS ONE. We would like to confirm that all data required to replicate the results of this study are fully available within the article itself and its Supporting Information files. Specifically, the dataset includes all individual values underlying the reported means, standard deviations, and graphical analyses, ensuring transparency and reproducibility. Therefore, no additional external data sources are required, and the current Data Availability Statement accurately reflects the dataset availability. Regarding the mention of Table 5, this was indeed a typographical error, as no Table 5 is included in the manuscript. This has now been corrected in the revised version. All changes made in the manuscript have been highlighted in yellow for the editor’s and reviewers’ convenience.

Additional Editor Comments:

Major revision is suggested by the reviewers for the manuscript.


[Note: HTML markup is below. Please do not edit.]

Reviewers' comments:

Reviewer's Responses to Questions

**Comments to the Author**

1. Is the manuscript technically sound, and do the data support the conclusions?

The manuscript must describe a technically sound piece of scientific research with data that supports the conclusions. Experiments must have been conducted rigorously, with appropriate controls, replication, and sample sizes. The conclusions must be drawn appropriately based on the data presented.

Reviewer #1: Yes

Reviewer #2: Yes

2. Has the statistical analysis been performed appropriately and rigorously?

Reviewer #1: N/A

Reviewer #2: Yes

3. Have the authors made all data underlying the findings in their manuscript fully available?

The [PLOS Data policy](http://track.editorialmanager.com/CL0/http:%2F%2Fwww.plosone.org%2Fstatic%2Fpolicies.action%23sharing/1/010f019d99287204-4dee9e64-f943-43b6-a230-7258b23cedfb-000000/NIU9PA5zd4EQwjtWOvQ1C42Sh_saEnfhyWUURtiJ-PU=258) requires authors to make all data underlying the findings described in their manuscript fully available without restriction, with rare exception (please refer to the Data Availability Statement in the manuscript PDF file). The data should be provided as part of the manuscript or its supporting information, or deposited to a public repository. For example, in addition to summary statistics, the data points behind means, medians and variance measures should be available. If there are restrictions on publicly sharing data—e.g. participant privacy or use of data from a third party—those must be specified.

Reviewer #1: Yes

Reviewer #2: Yes

4. Is the manuscript presented in an intelligible fashion and written in standard English?

PLOS ONE does not copyedit accepted manuscripts, so the language in submitted articles must be clear, correct, and unambiguous. Any typographical or grammatical errors should be corrected at revision, so please note any specific errors here.

Reviewer #1: Yes

Reviewer #2: Yes

5. Review Comments to the Author

Please use the space provided to explain your answers to the questions above. You may also include additional comments for the author, including concerns about dual publication, research ethics, or publication ethics. (Please upload your review as an attachment if it exceeds 20,000 characters)

Reviewer #1:

1. The novelty should be more precisely defined. The main contribution is the combined assessment of multiple surface treatments with two resin-cement viscosities on the fatigue biaxial strength of 4YSZ, rather than focusing solely on surface treatment or zirconia bonding in general.
2. The experimental design is interesting, but the use of only two monotonic specimens per condition to define the fatigue parameters is a clear limitation.
3. A stepwise fatigue test involving 10,000 cycles at 20 Hz is effective for initial screening; however, it does not accurately mimic long-term clinical fatigue as constant-amplitude, high-cycle fatigue protocols do.
4. The manuscript could improve by clearly explaining the reasons behind selecting 75 MPa as the initial fatigue stress and the use of 25 MPa increments. While these choices seem to be derived from the monotonic test, the rationale should be articulated more explicitly.
5. The method for determining cement layer thickness has certain limitations. Using a digital caliper to measure thickness indirectly—by subtracting pre- and post-cementation measurements—may not achieve the precision required for a nominal layer of approximately 50 µm, particularly when this variable is a key part of the interpretation.
6. The analysis of the interface that led to the conclusion of 'no viscosity effect' is too limited. The SEM assessment of cement penetration was only conducted for H-CTRL and L-CTRL, making it insufficient to assume that all treated groups exhibit the same interfacial behavior.

1. We thank the reviewer for this important comment. The manuscript has been revised to better clarify its novelty. We agree that the main contribution of this study is not the isolated evaluation of surface treatments or resin cement viscosity, but rather the combined effect of multiple surface treatments and different resin cement viscosities on the fatigue behavior of 4YSZ ceramics. In addition, the novelty is reinforced by the fact that 4YSZ presents a distinct microstructural behavior compared to conventional zirconia (3Y-TZP), mainly due to its higher cubic phase content and reduced transformation toughening capacity. Although these differences have been previously described in the literature since the introduction of 4YSZ, several studies have highlighted its distinct mechanical behavior, particularly in relation to surface treatments. In this context, air abrasion, although widely recommended, may introduce surface defects that act as stress concentrators and contribute to crack initiation and propagation, ultimately affecting fatigue performance. Despite this, there is still a lack of studies investigating how different surface treatments interact with resin cement viscosity for this material. This aspect is particularly relevant because resin cement viscosity may influence its ability to penetrate and adapt to surface irregularities created by different treatments, potentially affecting stress distribution and fatigue behavior. Therefore, the interaction between surface topography and cement properties in 4YSZ may differ from previously reported systems. The Introduction section has been revised to better emphasize this combined and material-specific approach. The changes are as follows: "It is essential to investigate how different surface treatments, which produce distinct surface characteristics, interact with resin cement viscosity to determine their combined effect on mechanical performance. The novelty of this study lies in the combined evaluation of multiple surface treatments and two resin cement viscosities on the fatigue biaxial flexural strength of 4YSZ. Unlike previous studies that have predominantly investigated surface treatments or resin cement viscosity separately, this work integrates both factors within the same experimental framework, allowing a direct assessment of their combined effect on mechanical performance. To the best of the authors’ knowledge, no previous study has evaluated the interaction between surface treatment and resin cement viscosity on the fatigue biaxial flexural strength of 4YSZ.”
2. We appreciate the reviewer’s observation. We acknowledge that the use of two specimens for the monotonic test represents a limitation. However, it is important to clarify that the monotonic test in this study was not intended for statistical comparison, but exclusively to provide reference values for defining the fatigue loading protocol. In this context, the monotonic test functions as a screening step, used to estimate the initial load levels to be applied during cyclic fatigue, rather than to generate inferential mechanical data. This approach is well established in the literature on dental materials, where preliminary monotonic results are commonly used to determine a percentage of the failure load for fatigue testing, ensuring that the cyclic loading remains within a clinically relevant and subcritical range. Therefore, the purpose of the monotonic test is methodological, supporting the design of the fatigue experiment, rather than serving as a primary outcome. Additionally, increasing the number of specimens for monotonic testing would not substantially impact the fatigue outcomes, as the fatigue behavior is predominantly influenced by the cyclic loading protocol and material response under repeated stress, which were evaluated with an adequate sample size. This limitation has now been explicitly acknowledged in the manuscript. This approach is consistent with the literature on dental materials, in which preliminary monotonic testing is commonly used to define fatigue parameters based on a percentage of the failure load. Although larger sample sizes may improve statistical reliability, previous studies have adopted relatively small sample sizes for monotonic tests, commonly around n ≈ 5, even when these values are subsequently used to establish fatigue loading conditions (DOI: 10.1016/j.jmbbm.2023.106338; 10.1590/1807-3107bor-2018.vol32.0053; 10.1007/s10266-025-01179-1; 10.1002/jbm.b.35657). This reinforces that monotonic testing in this context is primarily exploratory, rather than inferential. Considering the large number of experimental groups included in the present study, the use of n= 2 per group is methodologically justified for this specific purpose, as it allows estimation of approximate failure loads without compromising the feasibility of the experimental design. Importantly, fatigue protocols are based on relative loading levels, and therefore do not require precise determination of absolute strength values at this stage. Additionally, increasing the number of specimens for monotonic testing would not substantially impact the fatigue outcomes, as fatigue behavior is predominantly governed by the cyclic loading protocol and the material response under repeated stress, which were evaluated using an adequate sample size.
3. We thank the reviewer for this relevant comment. We agree that stepwise fatigue testing does not replicate long-term clinical fatigue conditions, particularly when compared to constant-amplitude, high-cycle protocols. This limitation is inherent to accelerated in vitro approaches and has been acknowledged in the revised manuscript. However, it is important to emphasize that the objective of the present study was not to simulate clinical longevity, but rather to compare the fatigue behavior of different experimental conditions under controlled and standardized loading. In this context, the step-stress approach provides a sensitive and time-efficient method to differentiate material performance, especially when multiple variables and experimental groups are involved. From a mechanistic standpoint, step-stress fatigue promotes progressive damage accumulation, including subcritical crack growth, by exposing the material to increasing stress levels over successive steps. This strategy enables the identification of critical stress thresholds and failure patterns that would be difficult to capture using constant-load fatigue within a feasible experimental timeframe. Therefore, while it does not reproduce clinical time scales, it is highly effective for ranking materials and identifying differences in fatigue resistance. Accelerated fatigue methods are widely reported in the literature, particularly when the aim is comparative analysis under controlled conditions within a feasible timeframe (DOI: 10.1111/jopr.13705; 10.1016/j.jmbbm.2022.105455; 10.1016/j.jmbbm.2022.105278; 10.1016/j.dental.2021.12.015; 10.1016/j.jmbbm.2023.105794; 10.1016/j.dental.2022.04.02; 10.1016/j.dental.2022.11.017). Within this framework, the use of fixed cycle increments (e.g., 10,000 cycles per step) and progressive load increases is consistent with established methodologies designed to efficiently induce failure and discriminate between groups. Importantly, the selection of 10,000 cycles per step should be understood as a methodological convention, rather than a physiological threshold. This interval provides a balance between enabling sufficient cyclic damage accumulation and maintaining a feasible experimental duration, as discussed by Velho et al. (2020). Furthermore, current fatigue guidelines for dental ceramics do not define specific parameters for step-stress protocols, but rather describe general fatigue mechanisms without prescribing standardized cycle numbers, according to Kelly et al. (2017). This indicates that such parameters are inherently flexible and should be adapted to the study design. Consistent with this, the literature demonstrates considerable variability in fatigue testing protocols, with no consensus regarding optimal cycle intervals or loading strategies, reinforcing that methodological consistency and reproducibility are key considerations, as highlighted in recent reviews (Velho et al., 2022). The testing frequency (20 Hz) reflects a balance between experimental efficiency and mechanical relevance and has been widely adopted in fatigue studies involving brittle dental materials. Experimental evidence specifically in zirconia demonstrates that increasing loading frequency can significantly reduce testing time without substantially altering fatigue behavior. In particular, studies comparing frequencies ranging from 2 Hz (physiological chewing simulation) to 20 Hz have shown that fatigue strength at 20 Hz does not significantly differ from that obtained at 2 Hz, while the amount of monoclinic phase remains unchanged. These findings indicate that frequencies up to 20 Hz can be used to accelerate fatigue testing without significantly affecting the underlying fatigue mechanisms, which remain governed by subcritical crack growth in polycrystalline ceramics. Taken together, these considerations support the use of step-stress fatigue as an accelerated comparative tool, rather than a predictive model of long-term clinical performance. Accordingly, the results should be interpreted within this framework, providing insight into relative material behavior under progressive loading conditions. To clarify this aspect, the following statement has been added to the Discussion section: "The results of this study should be interpreted within the limitations of an in vitro design. Although the step-stress fatigue approach is effective for comparative analysis, it does not fully replicate long-term clinical conditions, and therefore, the findings should not be directly extrapolated to predict clinical longevity.” (DOI: 10.1016/j.jmbbm.2020.103905; 10.1016/j.dental.2017.09.006; 10.1016/j.jmbbm.2021.104985; http://dx.doi.org/10.1016/j.jmbbm.2016.01.008).
4. We appreciate this comment and agree that further clarification improves transparency. The initial stress level of 75 MPa was defined based on the results of the monotonic test and corresponds to approximately 15% of the mean flexural strength, which is consistent with commonly adopted strategies in fatigue testing of dental ceramics. This approach ensures that loading begins within a subcritical stress range, allowing the accumulation of damage without immediate catastrophic failure. The increment of 25 MPa (≈5% of the mean strength) was selected to provide a gradual and controlled increase in stress, enabling sufficient sensitivity to detect differences in fatigue behavior between groups while maintaining experimental efficiency. Smaller increments improve the resolution of failure thresholds, which is particularly relevant in step-stress fatigue protocols.

This methodology is consistent with previously published studies that have adopted similar percentage-based approaches to define initial stress levels and incremental loading steps (DOI: 10.1016/j.jmbbm.2023.106338; 10.1590/1807-3107bor-2018.vol32.0053; 10.1007/s10266-025-01179-1; 10.1002/jbm.b.35657), reinforcing that these parameters are based on relative mechanical behavior rather than absolute values.

5. We thank the reviewer for raising this important point. Although indirect measurement using a digital caliper may have limitations for precise quantification of thin layers, it is important to clarify that the purpose of this procedure was not to determine absolute thickness values. Instead, it was used as a control measure to ensure standardization among specimens and to exclude those outside the target range (~50 µm). Thus, the method was adequate for its intended purpose, namely maintaining consistency across groups rather than providing high-resolution thickness measurements. Importantly, previous studies that measured the thickness of similar thin layers have employed the same indirect approach using a digital caliper (DOI: 10.1016/j.jmbbm.2023.106233; 10.1016/j.jmbbm.2023.105829; 10.1002/jbmb.70028), supporting the methodological validity of this procedure. Additionally, all specimens were fabricated under strictly controlled and standardized conditions, further minimizing variability and supporting the reliability of the adopted method.
6. We appreciate this observation. However, it is important to clarify that the SEM analysis was not intended to compare interfacial behavior across different surface treatments. Instead, its purpose was to provide a qualitative assessment of cement penetration and adaptation between the two viscosities (high and low) under a controlled condition (CTRL group). This approach was specifically adopted to reflect the fatigue findings of the present study, in which no statistically significant differences were observed between high- and low-viscosity cements. By isolating the control condition, the analysis allows a direct evaluation of viscosity without the influence of surface treatments, serving as a visual and qualitative support for the mechanical results. Therefore, the selection of H-CTRL and L-CTRL groups was intentional and methodologically justified. The absence of observable differences in interfacial behavior between viscosities is consistent with the fatigue outcomes, reinforcing the interpretation that viscosity did not influence the fatigue performance under the conditions tested. This rationale has been clarified in the manuscript to avoid misinterpretation.

Reviewer #2:

The manuscript presents an experimental investigation on the fatigue behavior of 4YSZ ceramics subjected to different surface treatments and resin cement viscosities. The topic is relevant to dental materials research, particularly for improving the mechanical reliability of zirconia-based restorations. The study is generally well-structured, and the experimental approach combining fatigue testing, surface characterization, and statistical analysis is appropriate. However, while the work provides useful insights, several methodological, analytical, and presentation-related issues limit its scientific rigor and broader impact.
Although, paper fits well to the submitted journal and, in general, it should be accepted for publication with following major revision suggestions:
1. The authors claim novelty in combining surface treatment with resin cement viscosity effects on 4YSZ. However, the incremental novelty is limited, as: Surface treatment effects on zirconia fatigue are already well established; The study confirms that viscosity has no significant effect, which aligns with existing literature.
2. The manuscript would benefit from clear positioning of its unique contribution, particularly: Why 4YSZ behaves differently from 3Y-TZP in this context; Whether the findings lead to clinically actionable recommendations.
3. The monotonic test sample size (n = 2) is insufficient for reliable estimation of fatigue parameters. This significantly weakens the reliability of the fatigue loading protocol derived from it. The fatigue test uses step-stress accelerated loading, but: No justification is provided for the chosen stress increments and cycle limits; The clinical relevance of 10,000 cycles per step is unclear.
4. The reported 100% statistical power appears unrealistic and likely results from: Overestimated effect size (f ≈ 1.5). Lack of clarity regarding: Whether assumptions for Two-way ANOVA are fully satisfied for fatigue data. Handling of censored data in survival analysis.
5. Weibull analysis is included but not sufficiently interpreted in terms of material reliability.
6. The discussion is largely descriptive and lacks deep mechanistic insight, particularly:
• No quantitative correlation between surface roughness (Ra/Rz) and fatigue strength.
• Limited discussion on crack initiation and propagation mechanisms in 4YSZ.
• The role of phase transformation (tetragonal → monoclinic) is mentioned but not experimentally supported.
7. Several grammatical and structural issues are present: Long sentences in the Introduction and Discussion reduce readability.
8. Some statements are repetitive (e.g., surface treatment dominance). Hypotheses should be more precisely defined.
9. Figures (SEM images) lack: Scale clarity in some cases. Quantitative annotations.
10. The manuscript relies heavily on prior zirconia studies but lacks: Discussion on recent advances in translucent zirconia systems. Comparison with alternative surface treatments (e.g., laser, plasma).

In conclusion, manuscript can be accepted after these suggested major revisions.

1. We thank the reviewer for this insightful comment and emphasize that the novelty of the present study is already explicitly stated in the manuscript, although its presentation can be further strengthened for clarity. As described in the Introduction, “to the best of the authors’ knowledge, no study to date has evaluated the effect of different resin cement viscosities combined with various surface treatments on 4YSZ,” which reinforces that the originality of this investigation does not reside in the isolated evaluation of surface treatments or resin cement viscosity, since both topics are well established in the literature, but rather in their combined and interactive assessment within a 4YSZ system under fatigue loading conditions, representing a clinically relevant and previously unexplored scenario. This approach is particularly justified from a biomaterials perspective because 4YSZ presents a fundamentally different microstructural behavior compared to conventional 3Y-TZP, characterized by increased cubic phase content, reduced transformation toughening capability, and altered crack propagation mechanisms, factors that directly influence the impact of surface defects and interfacial conditions on fatigue performance, which also means that findings derived from 3Y-TZP cannot be directly extrapolated to this material. In this context, the role of surface treatments in translucent zirconia’s is not fully established, and previous evidence indicates that airborne-particle abrasion may impair its mechanical behavior by introducing critical surface flaws, especially in materials more susceptible to defect-driven crack initiation. Cadore-Rodrigues et al. (2021) demonstrated that air abrasion increases surface irregularities and promotes stress concentration sites, which can reduce fatigue resistance in translucent zirconias, an effect that is particularly relevant for 4YSZ due to its limited ability to arrest crack propagation. In addition, a recent systematic review by Aurelio et al. (2016) reported that the current evidence regarding the influence of different air-abrasion particle sizes on the mechanical behavior of 4YSZ remains inconclusive, reinforcing that the response of this material to surface modification is still not fully understood. Furthermore, although previous studies such as Aragonez et al. (2023) and Packaeser et al. (2023) reported no significant influence of resin cement viscosity under specific conditions primarily limited to air abrasion, the present study advances the current knowledge by demonstrating that this absence of effect persists even across markedly distinct surface topographies, including both defect healing (GLZ) and defect inducing (AB110) conditions, which strengthens the robustness and generalizability of this finding and highlights that the interaction between surface condition and cement properties in 4YSZ remains an unexplored and clinically relevant aspect under fatigue conditions (DOI: 10.1016/j.jmbbm.2021.104543; 10.1016/j.dental.2016.03.021; 10.1016/j.jmbbm.2023.106233; 10.1016/j.jmbbm.2023.105829).
2. We thank the reviewer for this important observation and emphasize that the distinction between 4YSZ and 3Y-TZP is already addressed in the manuscript, as clearly presented in the Introduction, which states that “the higher cubic content limits tetragonal-to-monoclinic phase transformation, reducing the material’s ability to resist crack propagation and potentially compromising the mechanical performance of 4YSZ restorations,” and further reinforced in the Discussion, where it is noted that “the increased cubic phase content and, consequently, the larger grain size in 4YSZ complicate the phase transformation mechanism, making the ceramic more susceptible to failure”; from a biomaterials perspective, this reduced transformation toughening indicates that surface-induced defects play a more critical role in 4YSZ than in 3Y-TZP, which directly supports the rationale for focusing on surface treatments and fatigue behavior; however, it is important to clarify that the present study was not designed to establish a direct comparison between 4YSZ and 3Y-TZP, since the hypotheses and objectives are specifically centered on understanding the mechanical behavior of 4YSZ as a distinct material system, and therefore the discussion intentionally emphasizes its unique microstructural characteristics rather than conducting a comparative analysis between zirconia generations, an approach that is methodologically justified given that 4YSZ presents a specific structural configuration requiring independent investigation, particularly regarding the influence of surface conditions on fatigue performance; furthermore, although this is an in vitro study, it is well established in dental research that such experimental designs provide controlled and reproducible conditions that support cautious clinical extrapolation of the findings, which is why the present manuscript adopts a balanced interpretation of the results to ensure that the conclusions remain clinically meaningful without exceeding the inherent limitations of the experimental model; in this context, the study demonstrates that surface treatment is the primary determinant of fatigue performance, that glaze acts as a defect healing strategy, and that resin cement viscosity does not significantly influence fatigue behavior, collectively contributing to a more precise and clinically relevant understanding of how to optimize the performance of 4YSZ restorations.
3. We appreciate the reviewer’s thoughtful comments regarding the monotonic sample size and the definition of fatigue parameters. First, regarding the monotonic sample size (n = 2), it is important to clarify that monotonic testing in this study was not intended for robust statistical inference, but rather as a screening step to estimate initial loading parameters for fatigue testing. This approach is consistent with the literature, in which monotonic tests are frequently used to determine baseline strength values that guide subsequent fatigue protocols. Although larger sample sizes may improve statistical reliability, several studies in dental materials have adopted relatively small sample sizes for monotonic tests, commonly around n = 5 (DOI: 10.1016/j.jmbbm.2023.106338; 10.1590/1807-3107bor-2018.vol32.0053; 10.1007/s10266-025-01179-1; 10.1002/jbm.b.35657), even when these values are subsequently used to define fatigue loading conditions. For example, previous investigations evaluating fatigue behavior of ceramic materials have used monotonic results obtained from n = 5 specimens to establish fatigue parameters, reinforcing that this stage is primarily exploratory rather than inferential . Considering the large number of experimental groups in the present study, the use of n= 2 per group is justified and methodologically acceptable for this specific purpose, as it allows identification of approximate failure loads without compromising the feasibility of the study. Importantly, this strategy aligns with the conceptual framework of fatigue testing, in which initial monotonic data are used to define relative loading levels rather than absolute strength values. As highlighted in the literature, fatigue protocols often rely on a percentage of monotonic strength or on stepwise loading strategies derived from preliminary data . Additionally, a priori power analysis demonstrated a statistical power of 100% for the fatigue outcomes, confirming that the study is adequately powered to detect differences in the primary results. Therefore, the limited monotonic sample size does not compromise the validity of the fatigue findings, which represent the main outcome of this investigation. Regarding the step-stress protocol, accelerated fatigue methods are widely used in dental materials research (DOI: 10.1111/jopr.13705; 10.1016/j.jmbbm.2022.105455; 10.1016/j.jmbbm.2022.105278; 10.1016/j.dental.2021.12.015; 10.1016/j.jmbbm.2023.105794; 10.1016/j.dental.2022.04.02; 10.1016/j.dental.2022.11.017), particularly when the objective is to compare groups under controlled conditions within a feasible experimental timeframe. In this context, the use of fixed cycle increments (e.g., 10,000 cycles per step) and progressive load increases is consistent with established step-stress methodologies designed to efficiently induce failure and differentiate material performance. Importantly, the selection of 10,000 cycles per step is not based on a specific physiological threshold, but rather represents a widely adopted experimental convention, providing a balance between allowing sufficient cyclic damage accumulation (e.g., subcritical crack growth) and maintaining a feasible experimental duration, according to Velho et al. (2020). Importantly, current fatigue guidelines for dental ceramics do not establish specific recommendations regarding the number of cycles per step in step-stress protocols. Instead, they describe general fatigue mechanisms and testing approaches without defining standardized parameters, according to Kelly et al. (2017). This reinforces that such variables are not governed by fixed rules. Notably, studies employing similar step-stress approaches have adopted comparable cycle intervals without providing a specific biological justification, indicating that these parameters are primarily defined by methodological consistency and reproducibility. In line with this, a recent scoping review demonstrated a high variability and lack of standardization in fatigue testing parameters, emphasizing that the absence of comparative evidence makes it difficult to recommend specific protocols and that these choices should be guided by the study design and objectives, according to Velho et al. (2022). Therefore, the adopted protocol should be interpreted within the framework of accelerated fatigue testing, in which the goal is to efficiently induce failure and enable discrimination among groups, rather than to directly reproduce clinical loading conditions. Currently, there is no evidence supporting a standardized or optimal number of cycles per step in step-stress fatigue protocols. In summary, the adopted approach is supported by the literature and does not weaken the reliability of the fatigue protocol or the conclusions drawn from it (DOI: 10.1016/j.jmbbm.2020.103905; 10.1016/j.dental.2017.09.006; 10.1016/j.jmbbm.2021.104985).
4. We thank the reviewer for this insightful comment. Regarding the reported statistical power (1–β = 1.00), this value reflects the large effect size observed for surface treatment, which was the main factor influencing the outcomes. The effect size (f = 1.503887) was calculated based on the flexural strength means and their corresponding standard deviations, and therefore represents the magnitude of the differences observed in the dataset. Importantly, similar high statistical power values have also been reported in previous in vitro studies conducted under controlled experimental conditions (DOI: 10.1016/j.jmbbm.2023.105794), supporting that this finding is not uncommon when strong effects and standardized samples are present. It is important to emphasize that the statistical power was calculated a posteriori, and thus should be interpreted as a reflection of the observed data rather than a design assumption. Regarding the use of ANOVA, this analysis was performed exclusively to detect differences between factors in continuous variables and was not used as the primary outcome for fatigue behavior. The main analysis of fatigue performance was conducted using survival analysis (Kaplan–Meier and Mantel–Cox tests), which is the most appropriate approach for this type of data and properly accounts for censored observations. Concerning statistical assumptions, although formal assumption testing was not the primary focus, the adopted approach is supported by the literature. According to Blanca et al. (2017), the ANOVA F-test is robust to moderate deviations from normality, particularly when homogeneity of variances and balanced group sizes are present, as in the present study. This condition (equal sample size per group) contributes to the validity of the parametric approach even under non-ideal distributional conditions. Additionally, the application of ANOVA for mechanical data under similar conditions has been reported in previous studies, even when normality assumptions were not explicitly described, reinforcing its practical robustness in experimental dental research (DOI: 10.1002/jbm.b.35657; 10.1016/j.jmbbm.2025.106891; 10.1016/j.jmbbm.2024.106557). Furthermore, it is worth noting that the data were obtained under a highly controlled in vitro environment with standardized specimens, which reduces variability and contributes to the consistency of the observed effects.
5. We thank the reviewer for this valuable suggestion and emphasize that the Weibull analysis was intentionally included to assess the reliability and variability of fatigue behavior, which is particularly relevant for brittle biomaterials such as zirconia, where failure is governed by flaw distribution and defect sensitivity. As reported in the manuscript, “the Weibull modulus showed statistically similar results, suggesting equivalent structural reliability among all the groups analyzed.” This finding indicates that the distribution of critical flaws was comparable across all experimental conditions, supporting the interpretation that the intrinsic variability of the material was not significantly altered by the different surface treatments. Consequently, the observed differences in fatigue performance should be attributed primarily to the severity and nature of surface induced defects rather than to changes in flaw population or structural heterogeneity. This distinction is essential from a mechanistic standpoint, as it reinforces that the treatments influenced the magnitude of defects acting as stress concentrators without affecting the overall reliability of the material. To further strengthen this interpretation and improve clarity, the manuscript has been revised to include the following statement. “Thus, while surface treatments influenced fatigue strength, the similar Weibull modulus values indicate that the reliability of the material remained consistent across groups, suggesting that failure was governed by defect severity rather than variability in flaw distribution.” Additionally, it is important to emphasize that survival analysis using Kaplan–Meier estimates represents the primary analytical approach for fatigue data in this study. The Kaplan–Meier method considers the progressive increase in load and number of cycles as time-dependent variables, accounting for cumulative damage until failure occurs. Originally proposed by Edward L. Kaplan and Paul Meier in 1958, this method has been widely extended beyond clinical research to engineering and materials science applications, particularly in scenarios involving time-to-failure outcomes. The use of the log-rank (Mantel–Cox) test allows global comparison between groups by evaluating differences across the entire survival curve, making it especially suitable for fatigue testing under step-stress conditions. In contrast, although Weibull analysis is widely used to describe slow crack growth and probabilistic failure in brittle materials, its interpretation must consider sample size and variability. In the present study, a sample size of n = 15 per group was used, which is within the range commonly considered acceptable for Weibull analysis. However, it is acknowledged that larger sample sizes (e.g., n ≈ 30) may provide greater sensitivity to detect subtle differences in Weibull parameters. Therefore, while the absence of statistically significant differences suggests comparable reliability among groups, this finding should also be interpreted in light of the inherent limitations of sample size in probabilistic analyses. Therefore, Weibull analysis should be interpreted as a complementary tool in this study, whereas the primary conclusions are based on survival analysis, which appropriately accounts for censored data (i.e., specimens that did not fail within the testing limits) and is widely accepted in fatigue studies of dental ceramics. The combined use of both approaches ensures a comprehensive and methodologically sound evaluation of fatigue behavior. To further improve transparency, the limitation has been revised in the manuscript as follows: “Additionally, although Weibull analysis was performed to assess material reliability using a sample size of n = 15 per group, which is considered acceptable, larger sample sizes (e.g., approximately 30 specimens per group) may increase the sensitivity of Weibull parameter estimation. Therefore, the absence of significant differences in Weibull modulus should be interpreted with caution and considered complementary to the survival analysis, which represents the primary outcome of this study.”
6. We appreciate this valuable comment and respectfully emphasize that mechanistic aspects are already addressed in the manuscript, particularly through SEM analysis and the interpretation of surface induced defects, as exemplified by the statement “cracks initiate at the intaglio surface and propagate through the surface of the 4YSZ, influenced by the surface morphology resulting from each surface treatment,” which directly supports the discussion of crack initiation and propagation mechanisms in this material system; in addition, the Discussion provides a mechanistic framework by explaining that AB110 produces deeper defects that act as stress concentrators, while GLZ behaves as a defect healing layer, and that 4YSZ presents limited transformation toughening, increasing its sensitivity to surface damage, which collectively explains the observed differences in fatigue behavior; regarding the absence of a quantitative correlation between surface roughness parameters such as Ra and Rz and fatigue strength, it is important to highlight that this relationship has already been extensively established in the literature (DOI: 10.1590/0103-6440202103951), including studies such as Ares et al., and therefore the present study focused on the mechanical outcomes rather than reproducing correlations that are already well documented, allowing a more targeted interpretation of fatigue performance; furthermore, we acknowledge that no direct phase transformation analysis was performed, which represents a limitation of the study, although the discussion is supported by well-established evidence on zirconia behavior, particularly concerning the reduced transformation capability of 4YSZ; to address this point with full transparency, the following sentence has been incorporated into the manuscript “An additional limitation lies in the absence of direct phase transformation analysis, as the mechanistic interpretation is based on previously established literature on zirconia microstructural behavior, including the relationship between surface roughness and mechanical performance.”
7. We thank the reviewer for this comment. The manuscript has been thoroughly revised to improve grammar, sentence structure, and overall readability, with particular attention given to the Introduction and Discussion sections, ensuring greater clarity, coherence, and consistency in the presentation of the scientific arguments In addition, the study hypotheses have been reformulated for improved precision and clarity, as follows: “The hypotheses formulated were: (1) Surface treatment affects the biaxial flexural strength of 4YSZ; (2) Resin cement viscosity affects the biaxial flexural strength of 4YSZ.”
8. We appreciate this observation. While the emphasis on surface treatment was intentional due to its strong influence on the results, the manuscript has been revised to reduce repetitive statements and improve overall readability and textual flow. In addition, the hypotheses were refined to enhance clarity and precision without altering their original scientific meaning, ensuring a more concise and objective presentation of the study rationale.
9. We thank the reviewer for this comment and acknowledge the importance of clear figure presentation. The SEM images were carefully reviewed to ensure that scale bars are present, clearly visible, and consistently reported in all figures, and adjustments were made where necessary to improve clarity and readability. Regarding quantitative annotations, it is important to clarify that the primary purpose of the SEM analysis in this study was qualitative, aiming to illustrate representative surface topographies and defect patterns associated with each treatment, rather than to provide quantitative measurements. This approach is consistent with the role of SEM imaging in supporting the mechanistic interpretation of fatigue behavior. Nevertheless, to improve figure interpretability, minor visual annotations (e.g., arrows indicating representative features) have been included where appropriate to guide the reader, without altering the qualitative nature of the analysis.
10. We thank the reviewer for this relevant comment and acknowledge the importance of discussing recent advances in translucent zirconia systems as well as alternative surface treatments. The manuscript has been revised to reinforce the discussion on translucent zirconia, particularly highlighting the specific microstructural characteristics and mechanical behavior of 4YSZ, which is the focus of the present investigation. Regarding alternative surface treatments, we respectfully clarify that these approaches were not included because the current literature presents inconsistent and often unfavorable outcomes for zirconia, particularly related to surface damage and potential mechanical degradation, which limits the strength of any mechanistic or clinical interpretation. Therefore, the present study intentionally focused on well established and clinically relevant treatments, selected based on robust prior evidence demonstrating consistent and predictable results. Air abrasion remains a widely accepted standard approach, and the selection of parameters was supported by previous studies and systematic evidence, including the review by Aurélio et al. 2016, which emphasizes the limited investigation of different particle types specifically for 4YSZ. In order to address this point with transparency, the following limitation has been added to the manuscript “A limitation of this study is that only selected surface treatments with well established clinical relevance were evaluated, and additional surface modification approaches were not investigated, which may limit the generalizability of the findings.”

6. PLOS authors have the option to publish the peer review history of their article ([what does this mean?](https://track.editorialmanager.com/CL0/https:%2F%2Fjournals.plos.org%2Fplosone%2Fs%2Feditorial-and-peer-review-process%23loc-peer-review-history/1/010f019d99287204-4dee9e64-f943-43b6-a230-7258b23cedfb-000000/gShhoer37u7J9ZUXTBKPTQMLeXUUhom-oHs5pKT_czw=258)). If published, this will include your full peer review and any attached files.


If you choose “no”, your identity will remain anonymous but your review may still be made public.


**Do you want your identity to be public for this peer review?** For information about this choice, including consent withdrawal, please see our [Privacy Policy](https://track.editorialmanager.com/CL0/https:%2F%2Fwww.plos.org%2Fprivacy-policy/1/010f019d99287204-4dee9e64-f943-43b6-a230-7258b23cedfb-000000/EefjOYtO6eqhSYQd_ho8udFS7ZMlzZQLOWslwNoTbM4=258).

Reviewer #1: No

Reviewer #2: **Yes:** Dr. Himanshu Pathak
